# Advancing education in interventional psychiatry: scoping review of simulation training and the future of virtual reality-based learning

**DOI:** 10.3389/fpsyt.2025.1432314

**Published:** 2025-05-12

**Authors:** Peter Giacobbe, Alice Tu, Lilia Kaustov, Stephen Choi, Julian Wiegelmann, Fahad Alam

**Affiliations:** ^1^ Department of Psychiatry, Sunnybrook Health Sciences Centre, Toronto, ON, Canada; ^2^ Harquail Centre for Neuromodulation, Sunnybrook Health Sciences Centre, Toronto, ON, Canada; ^3^ Department of Psychiatry, University of Toronto, Toronto, ON, Canada; ^4^ Department of Anesthesia, Sunnybrook Health Sciences Centre, Toronto, ON, Canada; ^5^ Perioperative Brain Health Centre, Sunnybrook Research Institute, Toronto, ON, Canada; ^6^ Department of Anesthesiology and Pain Medicine, University of Toronto, Toronto, ON, Canada

**Keywords:** simulation-based learning, virtual reality, interventional psychiatry, neuromodulation, ECT, rTMS

## Abstract

**Objectives:**

Interventional psychiatric procedures such as electroconvulsive therapy (ECT) and repetitive transcranial magnetic stimulation (rTMS) have become increasingly important therapeutic options for managing severe or treatment-resistant mental illnesses. However, research suggests that gaps in training students in these techniques represent a rate-limiting step for their further dissemination and accessibility for the public. Studies have shown that the majority of psychiatry residents lack necessary competency and self-confidence in performing these treatments. Simulation based training has served as a gold standard for training procedural skills in medicine. Simulation-based training environments, particularly immersive reality technology (e.g., virtual reality [VR]), represent a promising novel avenue for trainees to develop the necessary skills for delivering these treatments. This scoping review discusses the current training in interventional psychiatry and how simulation-based training, specifically VR, can improve pedagogy in this area.

**Methods:**

In this scoping review, a literature search was conducted on the PubMed database using specific search terms such as “simulat*”, “training”, “ECT”, “TMS”, “neuromodulation”, and “interventional psychiatry”. The search was limited to studies with language in English from 1980 to 2023.

**Results:**

The initial search yielded 2094 articles, of which 4 evaluated the effectiveness of simulation approaches for ECT and were included in this review. No published studies were identified regarding VR-based education in ECT or rTMS.

**Conclusions:**

This scoping review provides an overview of the current landscape of pedagogical methods in interventional psychiatry and highlights the identified gaps in both the existing literature and the potential application of simulation-based environments, including VR, within this field. Considering the ongoing shift in medical education towards competency-based training, this review discusses the needs and benefits of VR-based simulators as an avenue to enhance competency in interventional psychiatry. Leveraging existing experience in the use of VR-based simulators in procedural skill acquisition in surgery and anesthesia, as well as recommendations on how to translate this approach to clinical training in psychiatry, are also discussed.

## 1 Introduction

### 1.1 The origins and scope of interventional psychiatry

With the continuing paradigmatic shift in the classification of mental disorders away from purely descriptive clinical phenomenology and towards emphasizing transdiagnostic biological systems in the brain underlying psychopathology (1), recent cohorts of psychiatry residents have expressed their interest in revamping clinical training to include wider integration of neuroscience-based education (2). The concept of interventional psychiatry was first described in 2014 to designate treatments for mental illness that are more procedural than general medical care within psychiatry and require specialized training in their delivery (3). Treatments that have traditionally been considered to fall under the rubric of interventional psychiatry have included brain stimulation treatments, such as electroconvulsive therapy (ECT) and repetitive transcranial magnetic stimulation (rTMS). ECT involves the application of an electrical current to the scalp to generate a therapeutic seizure under general anesthesia, whereas rTMS is a non-convulsive treatment that through the principle of electromagnetic induction, produces transient electrical currents in underlying cortical tissue, to either stimulate or inhibit brain activity, depending on the stimulation intensity and frequency (4). Both have proven antidepressant effects compared to sham (5), have first-line evidence as treatments for Major Depressive Disorder (MDD) (6), and have comparable positive effects on self-reported functioning in patients with treatment-resistant forms of depression (7).

The origins of interventional psychiatry have analogues in interventional cardiology and radiology, which both evolved over time through the consistent application of innovative technology to enhance patient outcomes (8, 9). Consistent with this, there has been interest in expanding interventional psychiatry beyond ECT and rTMS treatments, to incorporate newer procedures and modalities which do not directly apply energy to the brain, such as intravenous (IV) ketamine and intranasal esketamine. In the context of establishing the scope and breadth of a training program in interventional psychiatry at the University of Toronto, a survey was distributed to academic psychiatrists, asking which treatments they felt should be taught under the auspices of a competency diploma in interventional psychiatry (10). Participants included those that provide ECT and/or rTMS (deemed “interventionalists”, n=27) and those who do not deliver procedural or device-based treatments in their practice (i.e., “non-interventionalists”, n=37). There was 100% agreement that a competency diploma in interventional psychiatry should provide training in ECT and rTMS, but the majority also felt that IV ketamine (85.9%) and intranasal esketamine (71.9%) should be included, with equivalent rates of agreement amongst interventionalists and non-interventionalists alike. Approximately 1 in 5 respondents (19%) believed that other approaches, most commonly psychiatric neurosurgeries such as deep brain stimulation (DBS), MRI-guided focused ultrasound (FUS), and vagus nerve stimulation (VNS), should also be included.

This survey suggests that amongst academic psychiatrists, including non-interventionalists, there is wide acceptance of the value of training in interventional psychiatry. It sets a training mandate to extend the scope of this field to include ketamine treatments that have similar peri-procedural monitoring requirements as ECT (11). This is especially important in light of recent evidence of the comparable acute antidepressant effects of ECT and IV ketamine (12).

Based on the results of the survey described above (10) indicating that the majority of respondents advocate that training in Interventional Psychiatry should encompass ECT, rTMS, IV ketamine and intranasal esketamine, the remainder of the paper will explore the role of training in these modalities as barriers to their utilization. The majority of the discussion will focus on ECT, which has nearly a century of use as an established treatment and published literature. The exploration of barriers to the use ECT may provide insights into modifiable factors that would allow improved training and greater accessibility and utilization of other newer and emerging interventional treatment approaches in Psychiatry.

### 1.2 Barriers to greater utilization of interventional psychiatry treatments: lessons learned from ECT

Despite the fact that ECT arguably still represents the one of the most effective antidepressant treatments available and that it is considered a bellwether measure of a nation’s degree of financial investment in mental healthcare (13), evidence suggests that it remains underutilized and its use is stagnant or declining globally. ECT is the most geographically accessible of the interventional psychiatry modalities in Canada, with 84% of the population being able to access it within a one-hour drive (14). However, its rates of use in clinical settings remain low. Kaster analyzed healthcare registry data from 2009 to 2017 for all psychiatric admissions in Ontario with a diagnosis of a major depressive episode and found that only 1 in 11 (9%) involved the use of ECT (15), despite depressive disorders being the dominant clinical indication for ECT (6). During the period of 1992 to 2004, Rapoport et al. reported that approximately 12 per 100,000 Ontarians received ECT per year, without any significant change in utilization over this period (16). Administrative data from Quebec reported a decline in ECT utilization between 1996 and 2013 (17). Similar trends have been reported in the United States (18). To the best of our knowledge, no data from public registries regarding rTMS, IV ketamine, and IV esketamine treatment utilization has been published to date.

A number of factors have been identified as limiting greater ECT utilization. These include social and economic variables related to the cost of its delivery and misconceptions about the procedure among both the public and medical community (10). However, it is the pedagogy in these procedures during psychiatric training that has consistently emerged as the most proximal modifiable factor contributing to the low rates of ECT utilization (10). In a nationwide survey of ECT practitioners in the United States, the lack of trained ECT practitioners was identified as the top barrier to the implementation of an ECT program (19). Education about ECT during clinical training reduces the stigma among psychiatrists and increases the likelihood of patients being referred for this procedure in the future, even if the practitioners do not deliver ECT themselves (20). Therefore, education in ECT, and by extension other interventional approaches, during the critical developmental window of residency training may serve the dual purposes of improving accessibility to these treatments through both enhanced clinician awareness of these options in refractory patients and increasing the number of psychiatrists able to deliver it.

### 1.3 Trends in ECT and rTMS training

Both the expressed wish by psychiatric residents for more prominent inclusion of training in interventional psychiatry in residency programs and the data reviewed above suggest that educational experiences during this period of residency training are essential in increasing the accessibility of these treatments for the public. However, the existing literature on the rates of clinical competency in the delivery of ECT and rTMS reported by psychiatry trainees suggests that current educational paradigms have been inadequate.

With regards to ECT, training programs in the United States and Canada have advocated for psychiatry residents to obtain knowledge on its indications and uses, but without any formalized requirement for procedural exposure or competency (21–23). In Canada, the majority of ECT programs do not have a formal teaching program and in the programs that did provide teaching, methods varied widely with the most common methods of pedagogy being didactic lecture and indirect observation of the procedure without experiential learning and supervision (23).

In a survey of the emerging psychiatric workforce in Canada, of 162 residents from across the country who attended a national review course in preparation for licensure, 91% agreed that familiarity with both the theory and administration of ECT should be required to become a psychiatrist. However, only 24% reported achieving this benchmark at the end of their training (24). Rates of self-reported competency in the administration of ECT have been largely unchanged in Canada over the last three decades, with a rate of 25% (25) reported in 1991 and 18% in 2002 (26).

The stagnant rates of ECT competency amongst trainees in Canada since the 1990s appear to be secondary to a lack of opportunities to perform the procedure during training, rather than a dearth of didactic lectures. In the survey described above, 86% reported adequate teaching in the theory of ECT during their curricula, whereas 69% reported adequate supervision in the procedural aspects of ECT (24). However, rates of self-reported competency were significantly increased in respondents who reported having the opportunity to deliver ECT on more than ten occasions during their training, providing empirical validation for the minimal training requirements that have been advocated in the US (21).

Beyond ECT, there are identified gaps in training for other interventional procedures in psychiatry. For rTMS, 57% of residents indicated that competency in the delivery of this procedure should be required for licensure as a psychiatrist, but only 3% reported achieving this by the end of their residency training (24). To our knowledge, rates of competency to deliver IV ketamine and intranasal esketamine have not yet been reported in the literature. There has been a proposal for a curriculum to provide multidisciplinary training in both non-invasive neurotechnologies and surgical approaches to neuropsychiatric illness such as VNS and DBS (27), although the efficacy of this pedagogical model remains to be evaluated.

In summary, current models of pedagogy in interventional psychiatry which have emphasized learning through didactic lectures and indirect observation of treatment delivery, have proven to be insufficient and are unlikely to be effective in further translating emerging research findings in this area to improve rates of utilization and accessibility for those that can benefit from these procedures. A promising avenue to increase the rates of skilled practitioners may be through evolving post-graduate curricula to introduce educational opportunities that change the focus to the development of procedural competency.

### 1.4 Pedagogy in interventional psychiatry: future opportunities

With the recognition of the important clinical role for interventional treatments in refractory psychiatric illness, a number of international groups are now advocating for formalized training in this subspecialty within psychiatry (28, 29). The International Federation of Clinical Neurophysiology envisions training programs matched to three distinct classes of trainees: 1) the technician who delivers the treatment to the patient, 2) the clinician who establishes the indication for the treatment and prescribes the appropriate protocol, and 3) the clinician-scientist who designs and provides oversight for a research study involving an interventional approach (28). In this model, while each class of trainee has its own set of core training competencies in line with the role they play in the treatment’s delivery, an individual practitioner may have more than one role. For example, a physician may be expected to be a principal investigator for a study protocol involving TMS or ECT, assess patients for the presence of clinical indications/contraindications for these treatments, and then directly deliver the treatments. As a result, there is a growing need for comprehensive and broad training in a wide variety of interventional treatments to ensure their safe and effective delivery (28).

Another promising change to potentially increase rates of competency in interventional psychiatry is the shift in medical education towards competency-based medical education (CBME) (30). The main tenet of CBME is its greater emphasis on the implementation of outcome-driven education and assessment (i.e., outputs) through a focus on demonstrable abilities and performance needed for clinical practice rather than the traditional time-based focus on training inputs (31). The CBME approach allows for the skills expected of a psychiatrist to be deconstructed into their smaller summative tasks or competencies, which trainees can master through continuous practice. Indeed, competencies to achieve core and advanced proficiency in the delivery of ECT and rTMS have been identified (28, 32, 33). For rTMS, these include basic operation of the TMS device (e.g., turning the machine on/off, attaching coils, setting stimulation parameters, proper coil placement to establish the resting motor threshold and localization of the therapeutic target for stimulation [e.g., the dorsolateral prefrontal cortex] using scalp markers or MRI-based neuronavigation) (28). ECT procedural competencies include selecting the appropriate electrical stimulus parameters, titrating the dose according to types of ECT, interpreting the adequacy of a seizure with clinical and EEG measures, and managing missed seizures and other complications (32). In summary, interventional psychiatry, by virtue of its treatments being composed of multiple sequential physical actions and tasks which can be easily demonstrated, observed and measured, naturally lends itself to a CBME approach for determination of competency.

Despite the enthusiasm regarding CBME in general, several questions remain regarding the implementation of an interventional psychiatry curriculum in this framework. The optimal timing of interventional training is unclear, although there are significant advantages in including it in the core training required by all residents (22). However, due to a scarcity of human and technological resources, many trainees in psychiatry programs may not have local access to the clinical supervision and procedural exposure which are required to develop expertise in these treatments. The majority of ECT programs in Canada do not have a formal teaching program for trainees and there is a lack of uniformity in pedagogical approaches in those that exist (23). In the US, a limited subset of residency programs (i.e., 12 out of over 200 existing programs) train a disproportionate number of ECT practitioners across the country (19). As a result, the use of alternative forms of learning need to be explored to enhance competency in interventional psychiatry procedures.

## 2 Simulation in interventional psychiatry pedagogy: a scoping review of the literature

Simulation in medical education is a well-established pedagogical practice that offers an alternative to practicing directly with real patients. Through simulation, medical trainees have the opportunity to hone their skills until they reach a defined level of competency.

Given the current challenges of existing medical education models in providing sufficient training in interventional psychiatry and the promise of increased utilization of simulation-based learning to address this gap, it is imperative to review the existing evidence in this area. As a critical part of CBME and a gold standard for training procedural skills, simulation-based education in interventional psychiatry allows trainees to improve fluency in delivering treatments.

### 2.1 Methods

To gain a comprehensive understanding of the current state of simulation-based training in interventional psychiatry, this scoping review implemented a search strategy using the PubMed database, including human studies conducted from 1980 to 2023. To ensure inclusivity, a search string with wide criteria was employed: (simulat*[Title/Abstract] OR training[Title/Abstract] OR education*[Title/Abstract]) AND (electroconvulsive[Title/Abstract] OR ECT[Title/Abstract] OR “transcranial magnetic stimulation”[Title/Abstract] OR TMS[Title/Abstract] OR “interventional psychiatry” [Title/Abstract] or neuromodulation[Title/Abstract]).

Articles that met the following criteria were considered eligible sources: 1) evaluated any types of simulation-based learning in the field of interventional psychiatry, 2) targeted healthcare providers/trainees as learners, 3) only publications in English were considered, and 4) reviews were excluded. References were identified and screened by team member (AT) by title and abstract. Studies that met the inclusion criteria were further extracted and full-texts were reviewed.

### 2.2 Results

**Figure 1:**
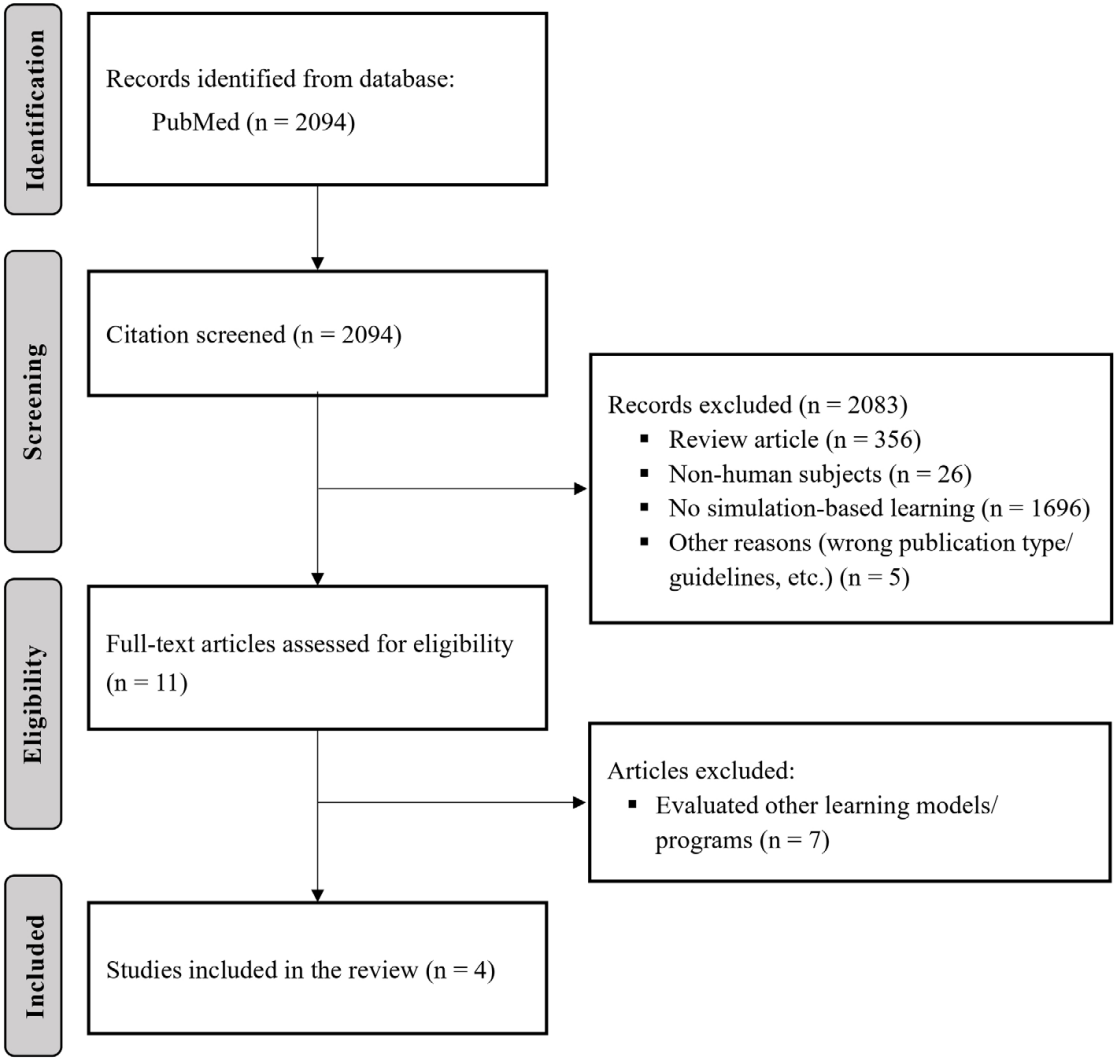
Flow diagram for literature reviews and retrieval.

The initial search yielded 2094 articles. After the pre-screening process based on titles and abstracts, 11 were sought for retrieval for full-text review, and 4 out of 11, which evaluated the effectiveness of simulation approaches for ECT, were included in this review. No published study was identified regarding simulation training for other interventional psychiatric treatments (e.g., rTMS) or VR-based education (**Figure 1**).

### 2.3 Review of the current use of simulation in ECT training

Based on our literature search, only 2 randomized controlled trials (RCT) (34, 35), 1 study reported in a letter to the editor (36), and 1 published protocol (37) were found regarding the evaluation of simulation training effectiveness for ECT.

The RCTs by Rabheru’s and Raysin’s groups utilized high-fidelity simulation (i.e., mannequin) as their simulation approach, which was compared to traditional didactics training. These two studies used ECT checklists to examine procedural skill competency, including electrode hook-up and placement, dosage strategies, procedure monitoring, etc.

In 2013, Rabheru et al. (34) were the first to investigate the effectiveness of simulation training on ECT clinical skills. Their study included 19 medical residents and assessed their performance on ECT skills and equipment use after receiving a high-fidelity patient simulator (n = 10) compared to didactic seminar training (traditional training; n = 9). In addition to procedural skills, they used standardized assessment scales such as Objective Structured Assessment of Technical Skills (ECT-OSATS) (38) to evaluate the residents’ knowledge and attitudes toward ECT. Their results revealed that although both groups showed similar increases in ECT knowledge and confidence, the residents who received simulator training demonstrated significantly better technical and overall skills compared to those who received didactics only (pass/fail ratio: 10/0 vs 1/8, χ^21^ = 15.53, *p* < 0.001).

In 2018, Raysin et al. (35) conducted an RCT comparing a control group of 17 residents who received standard didactic ECT curriculum with a simulation group of 18 residents who received augmented training with high-fidelity simulation in addition to didactics. In their study, a self-created experiential survey-cum-written test was administered to assess the residents’ overall experience with ECT (e.g., procedural knowledge, comfort level with the procedure) in addition to the skills evaluation. The study showed that the simulation group exhibited greater improvements in hands-on proficiency (pre-test to post-test mean scores: 22% to 51%, *p* < 0.001) compared to the control group (pre-test to post-test mean scores: 22% to 37%, *p* = 0.001). Differing from Rabheru’s study, their simulation group also demonstrated a significant increase in knowledge gain (pre- to post-test mean scores: 51% to 69%, *p* = 0.02), while the control group did not (*p* = 0.2). Higher comfort levels and confidence improvements in delivering ECT were also observed in Raysin’s study in only residents who had received simulation training.

In addition, Daaboul et al. (36) reported their findings from a high-fidelity simulation program in a letter to the editor. The study team used the Attitudes component of the Questionnaire on Attitudes and Knowledge of ECT (39) to assess a total of 31 psychiatry residents’ attitudes toward ECT before and after the program, with higher score indicating more negative attitudes. They found that after the high-fidelity simulation program, there was a significant decrease of 4.1 points (*p* < 0.001, t = 5.84) in the Attitudes score, suggesting the residents’ improvement in positive representations and attitude toward ECT.

In 2020, Aakhus et al. presented a research protocol (SAFE-ECT) for an ECT simulation program using a VR platform (37). VR-based simulation for surgical skills has become popular in recent years, and Aakhus’s team developed a checklist-based simulation training program for ECT using VR. They designed a prototype using the Unity3D graphic motor with the VR Goggles Oculus Rift, enabling users to repeatedly practice ECT skills step-by-step based on a standardized checklist. Their future plans included adding in different clinical scenarios and assessing the feasibility and applicability of the program in practice settings. While the efficacy of this particular VR-based simulation program for procedural skill acquisition have not yet been reported, if validated, it represents a promising direction for ECT training.

Reflecting on the studies reviewed above, there is limited but encouraging evidence supporting the effectiveness of simulation-based training in enhancing competency in interventional psychiatry, especially for ECT procedural skills. Adopting a simulation-based approach could be a viable solution to address the issue of stagnancy in the development of competency among psychiatry residents to perform these procedures. Furthermore, exploring novel simulation modalities, such as VR-based technology, could provide trainees with unlimited access to practice the logical steps of the treatments before real-life applications, thereby improving trainees’ confidence and enhancing patient safety. Offering this cost-effective and accessible alternative has the potential to expand the capabilities of simulation training in interventional psychiatry irrespective of geographical distance and other restrictions (e.g., remote areas, pandemic) where traditional simulation methods may pose challenges.

## 3 Immersive reality in medical education: lessons learned from other medical specialties

The current common approach in medical education is mannequin-based simulation requiring highly qualified personnel, equipment, and dedicated space. These factors contribute to high costs associated with high-fidelity medical simulation and also limit access to learners who are able to access these facilities in person for training and continuing education (40).

Accessibility to training is an even greater challenge for trainees in low and middle income countries (LMIC). In a survey conducted on psychiatric training among members of associations of the World Psychiatric Association, wide disparities were seen in the quantity and quality of psychiatric training worldwide, with educational limitations faced by LMIC countries identified as a primary factor (41). Trainees in LMIC countries had significantly less time dedicated to their psychiatric training (a mean of 39.3 months vs. a mean of 56.3 months in high-income countries) (41) and faced greater resource constraints in terms of accessibility to clinical supervisors, as well as ongoing teaching and training (42). With increased internet access in LMICs, many respondents expressed a preference for online training and remote learning to augment the development of skills and knowledge, including simulation learning through technologies such as IR (41, 42). As the cost of technology continues to drop and accessibility grows with each generation of commercially available devices, the role of IR simulation as a pedagogical technique to address gaps in training in psychiatry becomes more prominent. IR simulation allows trainees to have unlimited and on-demand training experiences. Thus, it may especially have a critical role in skill acquisition for interventional psychiatry procedures in locations where access to skilled supervisors is limited. Additionally, pursuing training in a home environment may be especially important in situations, where the ability to learn procedures through iterative face-to-face contact with patients is restricted.

The concept of IR in the healthcare realm is not new. IR is an umbrella term that includes VR, any type of interactive, computer-generated immersive learning element, and augmented reality, which involves overlaying computer-generated elements over a real-life environment (43). IR simulation can be an effective and efficient teaching method and in some cases, more effective than, traditional methods in medical education (44). IR offers several potential advantages, including increased accessibility (learners can use the equipment on-site or access IR educational applications remotely) and easy-to-set-up equipment in small spaces, requiring minimal time. This potentially allows for just in time curriculum delivery in multiple clinical settings. While there are initial costs associated with developing IR applications, the end products can be designed as modular systems with long term cost savings (45). Unlike mannequin-based curriculums, costs can be minimized for IR curriculum delivery and can be scaled for additional learners. These advantages offer the feasibility of a wide range of procedures, virtual patient interaction, and the potential for reduced costs in IR-based simulation training, although the validation of these benefits would still require further well-designed cost-benefit studies.

Educators in acute procedural medical specialties are faced with the challenge of balancing trainee autonomy with reduced clinical exposure. Learning technical skills through passive means, such as reading written text or watching it be done, has not been viewed as the ideal learning pedagogical approach in this area (46, 47). IR technology moves pedagogy by away from traditional methods of learning through two-dimensional videos and images to interactive simulated environments (48). IR allows learners to not only visualize procedural skills from a first-person perspective but also actively practice the required steps needed for completion in an active manner, with or without haptic capabilities. Even though haptic glove technology is still evolving and has not made it to the mainstream commercial market, the sense of touch can be simulated using commercial videogame joysticks, and common sensors attached to common medical tools (e.g., syringes, laryngoscope) (49, 50). These features may be especially important in medical education in interventional psychiatry, where the required knowledge acquisition and consolidation of procedural skills demand experiential, self-directed, and hands-on learning.

IR in procedural skill acquisition offers trainees a safe, realistic, and engaging learning environment. It can provide customized interactive case scenarios, repetitive training, emotional engagement, critical thinking, and immediate feedback to enhance learning outcomes. These interactive virtual curricula can be integrated into educational learning programs to train students, nurses, and doctors. Indeed, a recent scoping review by Mergen et al. reported the various uses and supported future efforts in VR development and its integration into medical education (51).

In the context of surgical subspecialties, IR simulators have been particularly beneficial for training surgical skills in laparoscopic, neuro, endovascular, and orthopedic surgeries (52–55). Anesthesia-related applications also benefit significantly from VR simulations. VR simulators can replicate a wide range of scenarios, teaching regional anesthesia techniques (56–59), bronchoscopy/intubation (60–64) and central vein insertion (65, 66). The development of IR training programs in these specialties have demonstrated positive learning outcomes, allowing trainees to navigate 3D anatomical structures and develop and refine procedural skills in a risk-free environment.

## 4 Discussion

In summary, there has been tremendous growth in the data supporting the use of interventional psychiatry techniques, especially in the last two decades. Both current trainees in psychiatry and established practitioners recognize the need for training in this area. However, the established models of medical pedagogy in psychiatry emphasizing knowledge acquisition through lecture and observation have been proven to be insufficient, and the majority of psychiatrists graduate without achieving competency in delivering ECT, the most established of the interventional psychiatry procedures. Education in interventional psychiatry during the critical developmental window of residency training may serve the dual purposes of enhancing clinicians’ awareness of these treatment options for refractory patients and increasing the number of psychiatrists able to deliver it. There is a need to shift the model of education in this area to reflect tenets of CBME, to focus on the outputs of training that are demonstrable abilities and performance of skills, rather than inputs or the traditional time-based focus in residency.

Considering the limited clinical exposure opportunities that currently exist for novice professionals and students, there is a need to explore alternative educational approaches that can provide enhanced training experiences in interventional psychiatry. There is currently a limited data on the use of simulation-based learning for interventional psychiatry treatments, but as demonstrated in other medical specialties, VR simulation training offers a safe, realistic, and interactive environment for students to practice complex procedures, develop critical decision-making skills, and improve patient safety. The use of VR-based simulator training by offering immersive, realistic, and on-demand training environments that can be accessed remotely, may play a role in addressing the low rates of competency to deliver interventional treatments by augmenting traditional models of pedagogy.

VR-based simulation training may also contribute to skill acquisition for students in locations where access to skilled supervisors is limited. As technology continues to advance and become more available worldwide, barriers to the deployment of VR simulation in interventional psychiatry will continue to decrease. Future research efforts will need to focus on investigating the effectiveness and practicality of these novel educational strategies.
